# Crisis Averted: Clinical T1b Renal Mass with Concurrent Arteriovenous Malformation and Renal Vein Thrombus

**DOI:** 10.1155/2022/9176199

**Published:** 2022-11-17

**Authors:** David Zekan, Kareem Wasef, Zachary Werner, Robert Grammer, Cara Lombard, Adam Luchey, Ali Hajiran

**Affiliations:** ^1^West Virginia University Department of Urology, 1 Medical Center Drive, Morgantown, WV 26506, USA; ^2^West Virginia University Department of Radiology, 1 Medical Center Drive, Morgantown, WV 26506, USA

## Abstract

Arteriovenous malformations (AVMs) secondary to renal-cell carcinoma (RCC) are well-described in the literature. Independently, renal vein and inferior vena cava tumor thrombi can be detected in locally-advanced RCC. A 67-year-old gentleman presented with a cT1b renal mass detected on workup for elevated creatinine. Multiphase CT imaging obtained for partial nephrectomy surgical-planning revealed an initially-missed renal cortical AVM. This drastically changed the plan for intervention, including use of an open approach with AVM embolization by interventional radiology prior and avoidance of a nephron-sparing approach. Final pathology confirmed the AVM and a subclinical renal vein thrombus masked by arterial flow on CT imaging, making this the first concurrent case described in the literature. Herein, we describe avoidance of catastrophic intraoperative hemorrhage by careful review of preoperative imaging and provide a literature review of imaging modalities for both renal surgical-planning and detection of tumor thrombi in RCC.

## 1. Introduction

In 2022, kidney cancer represents 4.1% of all adult malignancies in the United States with clear-cell renal-cell carcinoma (RCC) being the most common primary malignancy of the kidney [1]. RCC comprises 90% of primary malignant renal-neoplasms in adults and accounted for approximately 2% of all cancer deaths in 2019 [2]. It is diagnosed almost two times more in men, most commonly between the ages of 60 and 70 years [3]. With a primarily expansile growth pattern, it can spread via direct extension following breach of the renal fascia. This leads to a predilection for RCC to compress and involve hilar structures, most commonly the renal vein. Right-sided tumors are more likely to involve the vena cava due to the right renal vein's proximity and short length. RCC tumor thrombus extension can lead to involvement of the hepatic veins, supraphrenic IVC, and right atrium [4]. However, over half of RCCs are asymptomatic and diagnosed during thoracoabdominal imaging obtained for an unrelated reason. Those that do present symptomatically no longer garner the classic triad of flank pain, hematuria, and palpable abdominal mass. Instead, hematuria is most commonly the sole presenting symptom [3].

Radical and partial nephrectomy are long-established treatment modalities for suspicious renal masses. It is critical for surgeons to obtain and review dedicated high-quality cross-sectional renal imaging for presurgical planning to optimize oncologic control and minimize intraoperative complications. CT is often used over MRI due to lower cost and greater availability, and arterial- and nephrogenic-phased are recommended. At least two-phase imaging can help with pathologic subtype differentiation. Three-phase imaging is more commonly being utilized for surgical planning. Extension of RCC into the renal vein and vena cava is detected in 25% and 10% of patients, respectively, and preoperative thrombus detection is crucial for determination of surgical approach [5].

Herein, we present a rare case of a 6 cm renal mass that initially appeared to be amenable to partial nephrectomy; however, additional dedicated imaging raised concern for the presence of a cortical arteriovenous malformation (AVM), which changed the patient's surgical plan, outcome, and histologic findings.

## 2. Case Presentation

**Figure 1:**
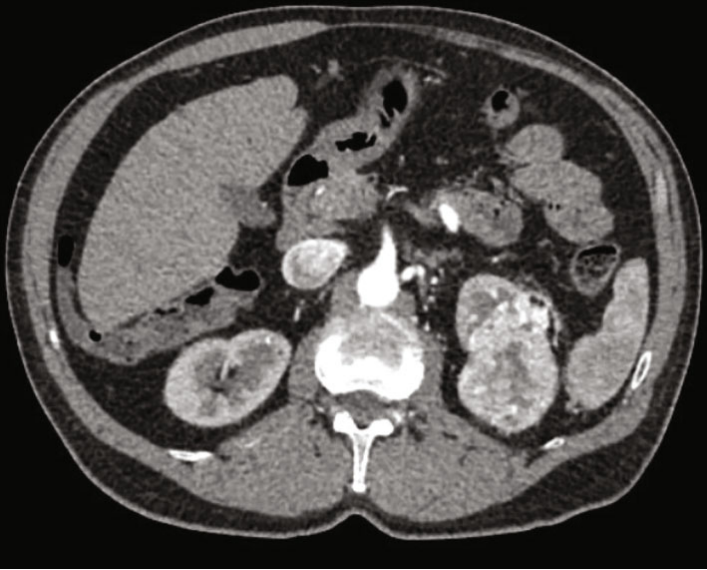
Axial CT scan showing posterior upper pole left enhancing renal mass concerning for malignancy.

**Figure 2:**
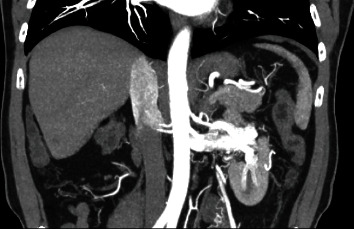
Coronal CT arterial phase showing two left renal arteries and early venous filling of contrast in renal vein and vena cava concerning for AVM in addition to multiple collateral vessels.

A 67-year-old otherwise healthy male presented with a left upper pole partially endophytic T1b renal mass diagnosed incidentally on imaging during workup for elevated creatinine. He was referred to our tertiary care center for consideration for a robotic partial nephrectomy. The differential diagnosis at this point included RCC, oncocytoma, or metastatic deposit from another primary cancer. The patient presented with a CT scan of the abdomen and pelvis with and without intravenous contrast that demonstrated the tumor and, per the radiology report, no evidence of vascular invasion on portal venous phase imaging (Figure 1). Upon further review of the imaging, there appeared to be abnormal enlargement of the renal vein, prompting additional investigation with a dedicated presurgical-multiphase CT scan with an arterial phase. Subsequent findings of early venous filling and multiple engorged collateral vessels with arterialized blood flow raised concern for the presence of a cortical AVM (Figure 2). These findings proved to be critical as the presence of AVM can lead to uncontrolled intraoperative hemorrhage during renal manipulation.

**Figure 3:**
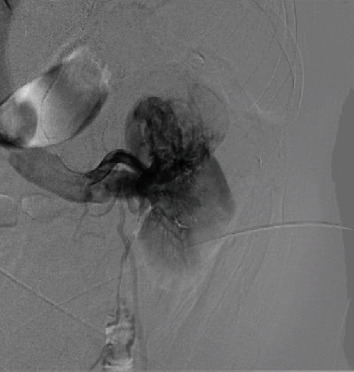
Selective angiography of main renal artery showing early filling of enlarged left renal vein with arterialized flow within the renal vein.

**Figure 4:**
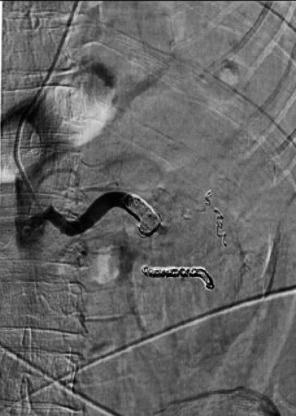
Status post selective coil embolization of the main renal artery at the level of the AVM with absence of early venous filling.

**Figure 5:**
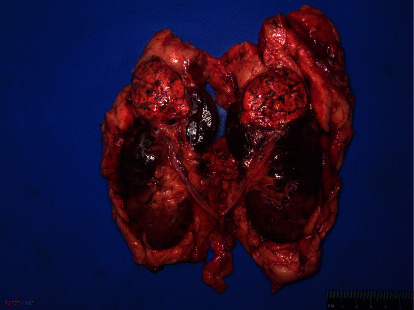
Gross pathology showing left kidney with upper pole renal mass, perinephric fat, and proximal ureter en bloc.

**Figure 6:**
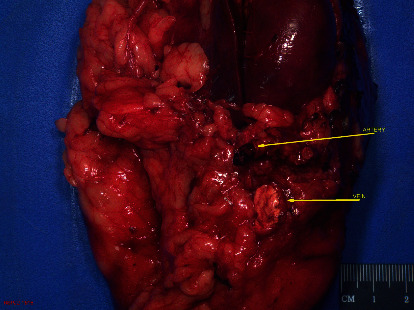
Gross pathology showing coil within the renal artery and tumor thrombus in the main renal vein.

The patient subsequently underwent a selective arterial angiography with embolization by interventional radiology (Figures 3 and 4) followed by an open radical nephrectomy within 24 hours. Intraoperatively, renal vein tumor thrombus invasion was found, which was masked by arterial flow from the AVM on imaging. The surgery was successful, and the patient recovered well without complication. Final pathology revealed clear-cell renal-cell carcinoma grade 2 with renal vein tumor thrombus, AVM within renal cortex, and tumor invasion of the renal sinus (pT3a) with negative surgical margins (Figures 5 and 6).

## 3. Discussion/Conclusion

AVMs arise from absence of a capillary bed resulting in aberrant shunting of blood from arterial to venous systems. Their prevalence in the general population has been approximated at 0.04% [6]. They can be congenital or acquired through trauma, biopsy, or malignancy. In the context of RCC, there have been 25 cases reported in the literature [7]. RCC can arise from VHL mutations and commonly produces high levels of angiogenic growth factor, contributing to highly vascular tumor presentations [8]. This likely contributes to their rare association with AVMs ([6, 9], and [10]). Renal AVMs can present similarly to RCC with hematuria and flank pain and can be challenging to differentiate on imaging. Indeed, there are reports of AVMs masking the presence of renal tumors on imaging [11]. Conversely, our case involved a renal mass with subsequently discovered AVM, which altered the surgical plan from partial to radical nephrectomy.

Perhaps, the largest alteration in AVM management in the past quarter century is diagnosis with contrasted CT and MRI, as opposed to direct angiography in the past [12]. Aberrant hilar vasculature or collateralization may arise following embolization. Sharma et al. described resection of a left-sided renal mass status post angioembolization characterized by multiple small arterial branches entering directly from the aorta and a large aneurysm at the junction of the renal and gonadal veins [13]. Resection of such a mass without anatomic delineation could lead to catastrophic hemorrhage. Likewise, attempted partial nephrectomy in the mass described is high risk based on both a potential for large arterial parasitic vessels when considering its relation to the renal hilum and a high pressure venous system due to aberrant arterial flow causing a potential for significant back-bleeding. One could encounter significant back-bleeding from the venous system which could compromise visualization and the ability to perform a proper partial nephrectomy without compromising oncologic control or having to convert to a radical nephrectomy. Also, arteriovenous malformations have a risk of spontaneous rupture, which could occur during renal mobilization while performing hilar dissection. In addition, in this case, flow from the arteriovenous malformation was masking a thrombus within the renal vein; so there is also a danger of proceeding with a partial nephrectomy or radical nephrectomy without preoperative angioembolization. An undiagnosed venous thrombus could contribute to a fatal embolic event and/or poor oncologic control, particularly in the setting of a high-pressure venous system. If partial nephrectomy were to be attempted in the setting of a single renal unit, preoperative anatomic delineation with a high-fidelity CT scan and intraoperative assurance of ischemia using indocyanine green as an immunofluophore would be crucial. Due to the above, an open surgical approach would be preferable, as more rapid hilar control and more reliable resection of venous thrombus in a known high-pressure system can be achieved, particularly when performed through a generous midline, hockey stick, or Makuuchi incision.

Less commonly, AVMs related to RCC can lead to appreciable systemic symptoms, such as high-output heart failure. This phenomenon was described as early as 1975, an era in which RCC was often advanced at the time of diagnosis. At that time, 30% of patients with clinically-significant fistulae presented with cardiovascular complaints, and 60% were found to have cardiovascular findings on workup, most commonly abdominal bruit (72%) [14]. AVM in the setting of a primary renal malignancy was recognized as a reversible cause of hypertension and heart failure [14]. More contemporary case reports confirm RCC as the most common source of tumor-related AVMs (22 of 30 cases in one review) [12]. An abdominal bruit, systolic murmur, and hypertension are present in over half of cases [12]. Tobe et al. described that right heart catheterization, when performed, consistently shows increased cardiac output and oxygen saturation levels, with improvement in both parameters postoperatively, indicating additional benefit gained beyond oncologic control.

Contrast-enhanced CT is the gold standard imaging modality for the detection and characterization of renal masses. Renal mass CT protocols generally consist of an unenhanced phase and three enhanced phases: corticomedullary, nephrogenic, and delayed excretory. Use of these phases is instrumental in characterizing cystic masses and can be predictive of an RCC histologic subtype. An arterial phase can provide helpful preoperative information regarding hilar anatomy, but can also highlight a potential tumor-related AVM [15]. Excretory-phase imaging highlights the tumor's relationship to the collecting system, which will also assist in surgical planning, particularly when attempting a nephron-sparing approach. Addition of three-dimensional volume rendering, multiplanar reformatting, and maximum intensity projection further clarify relationships between hilar structures and tumor and have decreased the need for diagnostic catheter angiography for the sole purpose of preoperative planning [16]. Catalano et al. describe the use of the three above phases with multidetector CT and a high-resolution protocol (one-millimeter-thick images) to correctly detect the presence and size of RCC in all studied patients. This protocol allowed for 100% diagnostic accuracy of calyceal infiltration and the presence and extent of renal vein/IVC thrombus in their cohort [17]. However, when contrasted CT scans are unavailable, clinicians should have a high suspicion for RCC when both homogenous and heterogenous renal masses are composed on noncalcific regions within 20-70 Houndsfield units (HU) [18]. Pooler et al. reviewed 193 cases of histologically-proven RCC on noncontrast CT and found that all malignancies fell within this attenuation range, defined as the “danger zone” [18]. Masses measuring less than 20 HU on noncontrast CT may still represent RCC but are generally heterogenous with irregular margins. In rare cases, particularly papillary tumors with lack of enhancement on contrasted CT, gadolinium-enhanced MRI can be beneficial in diagnosing RCC if clinical suspicion is high [19].

Herein, we present the first reported case of concurrent renal vein thrombus and AVM in the setting of clinically-localized RCC. Although Harada et al. reported a case of a 50-year-old female presenting with a 1 cm right-sided renal AVM with subsequent embolization and nephrectomy, final pathology revealed no evidence of malignancy [20]. However, this case is similar to our own in that it demonstrated the utility of multiphase CT imaging in surgical planning for preoperative planning and prevention of catastrophic tumor thrombus embolization or hemorrhage following hilar ligation or tumor resection. In our case, specifically, had a partial nephrectomy been attempted without further imaging, it could have led to uncontrolled bleeding as well as poor oncologic control due to masked renal vein tumor thrombus. AVM identification would also be crucial in this case if renal mass biopsy were proposed, as aberrant flow increases venous pressure, increasing the likelihood of significant perihilar or subcapsular hematoma and significant hemorrhage.

In summary, the rare association of RCC with AVMs is important to recognize for patient safety and surgical planning. Because of their similarity in presentation, a high index of suspicion is needed to recognize these potentially coexisting lesions. Additionally, the presence of a venous thrombus invasion masked by an AVM should be considered, along with MRI to characterize the extent of thrombus. A radical nephrectomy after embolization is a safe and definitive treatment in the case of RCC presenting with an AVM.

## Data Availability

Further data regarding the imaging included in this study and other de-identified information pertinent to the case may be furnished by contacting the corresponding author.
